# Characteristics and clinical outcomes of adolescents infected by SARS-CoV-2: a systematic review

**DOI:** 10.1590/1984-0462/2024/42/2022241

**Published:** 2023-10-23

**Authors:** Cezenário Gonçalves Campos, Luciene Aparecida Muniz, Vinícius Silva Belo, Cássia Cristina Costa, Juscelino de Souza Borges, Rommel Larcher Rachid Novais, Fernanda Lívia Dutra Rabelo, Charles Henrique Martins, Márcia Christina Caetano Romano, Joel Alves Lamounier

**Affiliations:** a Universidade Federal de São João del-Rei, Divinópolis, MG, Brazil.; b Instituto Federal de Educação, Ciência e Tecnologia do Ceará, Limoeiro do Norte, CE, Brazil.; c Centro Federal de Educação Tecnológica de Minas Gerais, Divinópolis, MG, Brazil.

**Keywords:** Adolescent, COVID-19, SARS-CoV-2 infection, Health profile, Adolescente, COVID-19, Infecção por SARS-CoV-2, Perfil de saúde

## Abstract

**Objective:**

To verify the COVID-19 clinical characteristics, associated comorbidities, and outcomes in adolescents.

**Data source:**

This is a systematic review study based on articles published between 2020 and 2022 in the United States National Library of Medicine - PubMed (MedLine), Virtual Health Library – VHL (LILACS), Science Direct, Web of Science, and Scopus (Elsevier) databases. The study was registered in the International Prospective Register of Systematic Reviews, under No. CRD42022309108.

**Data synthesis:**

A total of 1188 studies were identified. After applying the selection criteria, 13 articles were included. Prevalence was 25%; mild cases were predominant; and fever, cough, headache, anosmia, nasal congestion, and ageusia were frequent. Fever and cough were proportionally higher in hospitalized cases: 81 and 68%, respectively. Dyspnea (odds ratio [OR] 6.3; confidence interval 95%[CI] 2.8–14.3), fever (OR 3.8; 95%CI 2.0–7.4), and cough (OR 3.4; 95%CI 2.0–6.0) were associated with severe cases. Up to 28% required intensive care and 38% required mechanical ventilation. Pre-existing comorbidities increased the risk of hospitalization and death. Severe cases were associated with the risk of death (relative risk [RR] 4.6; 95%CI 2.8–7.5). The black, mixed, and indigenous races/skin colors represented risk groups, as well as residents of poorer regions.

**Conclusions:**

The review provided a better understanding of the disease profile and may favor the development of public policies, in addition to contributing to the current literature in the field of adolescent health.

## RESUMO

**Objetivo:**

Verificar as características clínicas, as comorbidades associadas e os desfechos da COVID-19 em adolescentes.

**Fontes de dados:**

Trata-se de uma revisão sistemática elaborada com base em artigos publicados entre 2020 e 2022 nas bases de dados United States National Library of Medicine (PubMed), Biblioteca Virtual em Saúde (BVS), Science Direct, Web of Science e Scopus. O estudo foi registrado no *International Prospective Register of Systematic Reviews*, n° CRD42022309108.

**Síntese dos dados:**

Foram identificados 1.188 estudos. Após a aplicação dos critérios de seleção, 13 artigos foram incluídos. A prevalência foi de 25%; os casos leves foram predominantes; e febre, tosse, cefaleia, anosmia, congestão nasal e ageusia foram frequentes. Febre e tosse foram proporcionalmente maiores em casos hospitalizados, com 81 e 68%, respectivamente. Dispneia (OR 6,3; IC95% 2,8–14,3), febre (OR 3,8; IC95% 2,0–7,4) e tosse (OR 3,4; IC95% 2,0–6,0) foram associados a casos graves. Até 28% necessitaram de cuidados intensivos e 38% de ventilação mecânica. A preexistência de comorbidade aumentou o risco de internação e óbito. Os casos graves estão associados ao risco de óbito (risco relativo — RR 4,6; IC95% 2,8–7,5). As raças/cores preta, parda e indígena e os moradores de regiões mais pobres foram grupo de risco.

**Conclusões:**

A revisão permitiu conhecer o perfil da doença e poderá favorecer a elaboração de políticas públicas, além de contribuir para a literatura atual no campo da saúde do adolescente.

## INTRODUCTION

In the general population, the clinical manifestations of COVID-19 are very heterogeneous, ranging from asymptomatic cases to severe respiratory conditions.^1,2^ Compared to adults and older adults, adolescents present milder manifestations.^2–5^ However, adolescents with chronic health conditions or who are immunosuppressed may develop severe acute respiratory syndrome (SARS). These conditions result in worse prognoses.^6,7^

Some countries in Europe and the United States estimate that the percentage of adolescents with a confirmed COVID-19 diagnosis ranges from 1 to 5% of the general population.^8–10^ However, this number can reach 25 to 30% in underdeveloped countries.^6,8,10^

Currently, the clinical characteristics and outcomes of the infection in adolescents are still unknown in many national and international territories.^1,5,6,10^ There is no consensus in the literature on this matter. The proportion of asymptomatic and symptomatic cases and the prevalent symptoms are not evident, nor are the prevalence and mortality, need for admission to the intensive care unit (ICU), use of mechanical ventilation (MV), and comparison of the infection with other age groups.^6,9,10^

Considering the existing gap, simultaneously with the significant volume of adolescents affected by the COVID-19 worldwide, the global number of deaths, a lethality rate between 4.5 and 37% due to complications of the disease, the low vaccination coverage and adherence in some countries, and the identification of new circulating variants, it is essential to know the profile of the infection in adolescents.^4,6–8,10^ Therefore, this article presents a systematic review aimed at verifying the COVID-19 clinical characteristics, associated comorbidities, and outcomes in adolescents.

## METHOD

This is a systematic literature review carried out on electronic databases to identify publications on COVID-19 clinical characteristics, associated comorbidities, and outcomes in adolescents. The study was registered in the International Prospective Register of Systematic Reviews (PROSPERO), under No. CRD42022309108.

The articles included were focused on adolescents aged 10 to 19 years, regardless of gender. Data collection occurred in April 2022, considering information since January 2020, and conducted by two independent researchers.

The electronic journal databases of the US National Library of Medicine of the National Institutes of Health (PubMed), Virtual Health Library (VHL), Science Direct, Web of Science, and Scopus were consulted. The Population, Exposure, Comparator, and Outcomes (PECO) strategy was used to elaborate the research question.^11,12^ The combinations of descriptors and keywords in English were constructed according to the Medical Subject Headings (MeSH): (*adolescent* OR *adolescents* OR *teenager* OR *teenagers*) AND (*COVID-19* OR “*COVID-19 Virus Disease*” OR “*COVID-19 Virus Infection*” OR “*Coronavirus Disease 19*” OR “*SARS-CoV-2 Infection*”) AND (“*signs and symptoms*” OR “*health profile*”).

Observational cross-sectional, case-control, or cohort studies were selected. Articles in English, Portuguese, and Spanish and those with access to full-text versions were eligible. For this review, the population aged less than 10 years or 20 years and older was not considered adolescent. Thus, our adolescent age group included patients from 10 to 19 years-old. Clinical trials, quasi-experimental studies, case studies, literature reviews, governmental documents, preprints, press releases, qualitative studies, and studies involving animals were excluded.

An initial screening was performed based on the titles and abstracts of all the articles found, in line with the inclusion and exclusion criteria and that answered some of the study questions; themes that were not consistent with the study were excluded, for example, those that dealt exclusively with cases and profile of pediatric multisystemic inflammatory syndrome or vaccines. The identification, screening, eligibility, and inclusion process of the articles found complied with the Preferred Reporting Items for Systematic Reviews and Meta-Analyses (PRISMA) protocol.^13^

The selection and evaluation of papers were autonomously conducted by two researchers (CG Campos and LA Muniz). The full texts were thoroughly reviewed, observing the criteria for inclusion. A database was created in Microsoft Word 2016, and the extracted variables were the following: reference, year of publication, country, study design, sample description, associated comorbidities, clinical characteristics, tests performed for diagnosis, outcomes, methodological quality, and level of evidence.

To assess the methodological quality of the articles selected, the questionnaire proposed by Downs and Black was used, consisting of 27 items in the form of questions, which cover the methodological evaluation of the studies, including internal validity, external validity, and statistical power. Five items of this instrument were not used, as they refer to experimental studies evaluation. Studies with a score of 16 points, that is, classification above 70% in the methodological evaluation, were included in the review.^12–14^ This evaluation was conducted by two independent researchers. Disagreements between them were resolved in a plenary session with a third researcher. The level of scientific evidence of the studies was classified according to the Agency for Healthcare Research and Quality (AHRQ) categorization.^12^

## RESULTS

**Figure 1:**
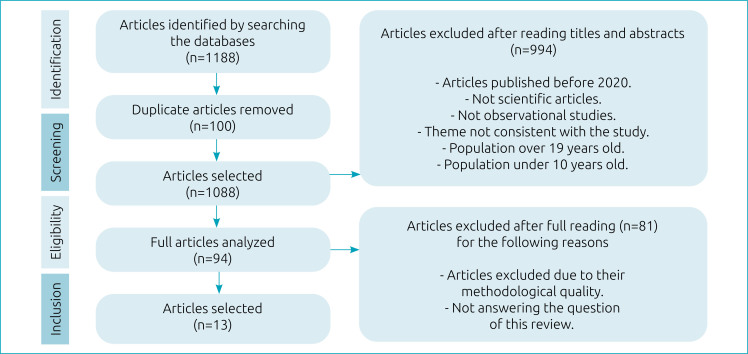
Research flowchart: identification, screening, eligibility, and inclusion of the scientific articles in the systematic review, according to PRISMA.^13^

When searching the databases, 1188 articles were found. Of this total, one hundred duplicate studies were removed, and after reading the titles and abstracts, another 994 were removed. The full reading was performed on 94 articles. Of them, 80 were excluded for not answering the question of this review and one for not reaching a score of 16 points in the questionnaire proposed by Downs and Black,^14^ yielding 13 studies included in the review (Figure 1).

The samples of the selected studies added up to 18,573 adolescents. Ten articles (77%) had cohort study designs and three were cross-sectional studies (23%). Two articles were published in 2020,^15,16^ ten in 2021,^17–26^ and one in 2022.^27^

Table 1 presents a description of the articles analyzed, published between January 2020 and April 2022; Table 2 shows the variables related to the associated comorbidities, clinical characteristics, and tests performed for the COVID-19 diagnosis; and Table 3 presents the outcomes found.

**Table 1:** Articles published between January 2020 and April 2022.

Reference	Country	Study design	Description of the sample Number of participants (n)/age group (years old)	Methodological quality^14^ and level of evidence^12^
Oliveira et al.^27^	Brazil	Cohort	n=6.725/12–19	17 points/level 3
Clemente et al.^19^	Spain	Cohort	n=77/less than 18	16 points/level 3
Drouin et al.^20^	Canada	Cohort	n=36/13–17	18 points/level 3
Gomes et al.^21^	Brazil	Cohort	n=1320/12–18	16 points/level 3
Pinto Júnior et al.^22^	Brazil	Cross-sectional	n=854/10–19	17 points/level 4
Graff et al.^23^	USA	Cohort	n=220/11–19	17 points/level 3
Macias-Parra et al.^24^	Mexico	Cross-sectional	n=34/12–18	16 points/level 4
Oliveira et al.^25^	Brazil	Cohort	n=3589/12–19	21 points/level 3
Parcha et al.^26^	USA	Cohort	n=5573/12–17	18 points/level 3
Prata-Barbosa et al.^15^	Brazil	Cohort	n=14/12–17	16 points/level 3
DeBiasi et al.^16^	USA	Cohort	n=73/10–19	16 points/level 3

**Table 2:** Associated comorbidities, clinical characteristics, and test performed for the COVID-19 diagnosis.

Variables		Frequencies or grouped frequencies
Associated comorbidities (%)		
	Neurological diseases	20
	Respiratory diseases	18
	Heart diseases	15
	Metabolic diseases	15
	Immunosuppression and malignancy diseases	15
	Kidney diseases	5
	Rheumatic and musculoskeletal and hematologic diseases	5
	Genetic syndromes	3
	Gastrointestinal diseases	3
	Dermatological diseases	1
Clinical characteristics (%)		
	Fever	11–81
	Cough	16–68
	Nasal congestion/Runny nose	18–63
	SpO_2_ * <95%	29–53
	Respiratory distress	30–52
	Tachypnea	10–50
	Anosmia	9–48
	Dyspnea	5–46
	Sore throat	9–44
	Gastrointestinal symptoms	4–42
	Headache	6–35
	Myalgia	7–14
	Ageusia	5–11
Diagnostic test (%)		
	Laboratory method for virus detection by RT-PCR†	65
	Viral immune response tests, antibody tests (serological tests)	15
	Rapid tests for detection of viral antigens	10
	Clinical-epidemiological method	10

* SpO_2_: peripheral oxygen saturation;

† RT-PCR: reverse transcription-polymerase chain reaction.

**Table 3:** Outcomes found in adolescents infected by SARS-CoV-2.

Reference	Outcomes
Oliveira et al.^27^	ICU admission rate (28%). Use of NIMV (38%) and IMV (22%). Lethality rate: 7.5%. Dyspnea (RR 2.1; 95%CI 1.8–2.4), SP0^2^<95% (RR 3.5; 95%CI 2.5–4.1), adolescents living in the North (RR 1.5; 95%CI 1.3–1.9) and Northeast (RR 2.0; 95%CI 1.7–2.4) regions, ethnic groups black, mixed-race (RR 1.3; 95%CI 1.1–1.5) and indigenous (RR 3.3; 95%CI 2.2–4.9) and the presence of comorbidity which increases the risk of death (RR 2.7; 95%CI 2.3–3.0).*
Afonso et al.^17^	Prevalence of SARS-CoV-2: 25% (95%CI 20.3–30.6). 55% were symptomatic. 17.7% had at least one type of comorbidity. Higher proportion of females (53.3%) and self-declared black or mixed-race (48%).†
Alharbi et al.^18^	Two participants were admitted to the ICU. One female, 12 years old, without comorbidities, no need of MV, hospitalized for 43 days, 14 in the ICU, and was discharged. The second adolescent developed P-MIS, he was male, 12 years old, neuropath, used MV, and died.*
Clemente et al.^19^	The median hospitalization time was five days (IQR: 2–20). One patient required ICU. There are no death records. Previous use of glucocorticoids is associated with a greater chance of hospitalization (OR 3.5; p=0.001). The comorbidities were not analyzed.*
Drouin et al.^20^	47% required hospitalization. Comorbidity is associated with severity (p=0.001). Hospitalizations were more frequent in patients with asthma (p=0.003) or metabolic disease (p=0.004). The severe form of the disease was frequent in patients with encephalopathy (p=0.005).‡
Gomes et al.^21^	Lethality rate: 13.6%. Risk of death: being an adolescent (RR: 1.6; 95%CI 1.1–2.3), SARS-critical (RR 4.6; 95%CI 2.8–7.5), immunosuppressed (RR 2.2; 95%CI 1.6–3.2).*
Pinto Júnior et al.^22^	29.3% of prevalence. 32% of cases presented comorbidities. 24% were symptomatic at the time of examination.†
Graff et al.^23^	45% presented comorbidities. The presence of comorbidities increases the chance of hospitalization (OR 2.7; p=0.003). The chances increase with each additional comorbidity (OR 1.4; p<0.001). Dyspnea (OR 6.3; 95%CI 2.8–14.3) is associated with chance of hospitalization and increases the chance of using MV (OR 15.7; 95%CI 6.4–38.5).*
Macias-Parra et al.^24^	23% required ICU admission. 17% required MV. 77% presented comorbidities (33% with chronic diseases and 44% with immunosuppressive and/or oncological diseases).*
Oliveira et al.^25^	The mean time from onset of symptoms to hospitalization was three days (IQR 1–6). 24% were admitted to the ICU. 10% required IMV. 7.5% died. Residents of the Northeast (RR 2.1; 95%CI 1.7–2.5), and North (RR 1.5; 95%CI 1.2–2.0), regions and indigenous ethnicity (RR 3.4; 95%CI 2.2–5.2), are at a higher risk of death.*
Parcha et al.^26^	5.5% were hospitalized. 1% required ICU. 0.3% MV. The hospitalization risk was higher in black (RR 2.0; 95%CI 1.5–2.6) and in Hispanics (RR 1.3; 95%CI 1.0–1.8).‡
Prata-Barbosa et al.^15^	All admitted to the ICU. One case developed P-MIS. One death of a female adolescent, 14 years old, with chronic liver disease. Presence of comorbidities is associated with disease severity and need for IMV (OR 5.5; 95%CI 1.4–21.1).*
DeBiasi et al.^16^	22% were hospitalized. 55% of those hospitalized presented no comorbidities. 25% of those hospitalized required ICU. There were no death records.‡

ICU: intensive care unit; NIMV: non-invasive mechanical ventilation; IMV: invasive mechanical ventilation; SpO_2_: peripheral oxygen saturation; CI: confidence interval; SARS-CoV-2: severe acute respiratory syndrome coronavirus 2; MV: mechanical ventilation; P-MIS: pediatric multisystemic inflammatory syndrome; IQR: interquartile range; OR: odds ratio; RR: relative risk; SARS-critical: severe acute respiratory syndrome presenting low oxygen saturation, respiratory distress, dyspnea and cyanosis.

* study carried out in a hospital environment;

† study carried out in a non-hospital environment;

‡ study carried out in a hospital and non-hospital environment.

In this research, two studies evaluated 903 adolescents in a non-hospital environment and found that mild COVID-19 cases were predominant.^17,22^ The highest proportion of the disease was observed in girls (53.2%) and in those with self-declared black or mixed race/skin color (48.3%); about 32% of infected adolescents had some comorbidity.^17,22^ The prevalence of SARS-CoV-2 was 25.1% (95%CI 20.3–30.6).^17,22^ Some 24 to 55% of cases were symptomatic, while anosmia (prevalence ratio [PR] 3.1; 95%CI 1.9–4.9), nasal congestion (PR 2.9; 95%CI 1.8–4.9), fever (PR 1.9; 95%CI 1.1–3.2), and ageusia (PR 1.8; 95%CI 1.1–2.8) were associated with COVID-19.^17,22^

Some 11 to 71% of the non-hospitalized adolescents reported fever, whereas, among hospitalized adolescents, the proportion ranged from 27 to 81%.^15,16,18–27^ The presence of cough varied from 7 to 51%, reaching 68.4% in severe cases.^15–17,19–27^ Headache was common in mild cases, with 11 to 55%.^17–22^ Gastrointestinal and musculoskeletal symptoms were less prevalent.^17,20,22,26^ Dyspnea (OR 6.3; 95%CI 2.8–14.3), fever (OR 3.8; 95%CI 2.0–7.4), and cough (OR 3.4; 95%CI 2.0–6.0) were associated with higher disease severity and increased chances of hospitalization, whereas the severe cases were associated with risk of death (RR 4.6; 95%CI 2.8–7.5).^21,23^

The lethality rate in hospitalized adolescents varied from 7.5 to 13.6%.^21,25,27^ The need for admission to an ICU ranged from 23 to 28.3%, the use of non-invasive MV from 17 to 37.6% and, in the invasive modality, from 10 to 22%.^16,24,25,27^ MV use was associated with signs of dyspnea (OR 15.7; 95%CI 6.4–38.5), fever (OR 5.3; 95%CI 2.2–12.5), and preexisting comorbidities (OR 5.5; 95%CI 1.4–21.1).^15,23^ Asthmatic adolescents were more likely to require MV (OR 3.1; 95%CI 1.4–6.9).^15^

Preexisting comorbidities increased the risk of hospitalization (OR 2.7; p=0.0003) and death (p=0.001).^23,27^ The chance of hospitalization was higher with each additional comorbidity (OR 1.4; p<0.0001).^23^ Obese adolescents were twice as likely to be hospitalized (OR 2.5; 95%CI 1.2–5.1), and the chance increased five times in cases of severe obesity (OR 4.8; 95%CI 1.9–12.1).^23^ The presence and number of comorbidities – one (RR 2.9; 95%CI 2.5–3.5), two (RR 4.9; 95%CI 3.8–6.5), three or more (RR 7.3; 95%CI 4.6–11.6) — were associated with death outcomes.^25^ The risk was higher in patients with asthma (p=0.03), epilepsy (p=0.03), chronic encephalopathy (p=0.005), obesity (p=0.04), and chronic lung disease (p=0.009).^20,21^ Similarly, immunosuppressed adolescents were at a higher risk of death (RR 2.2; 95%CI 1.6–3.2).^20,21^

Regarding the ethnic-racial characteristics, North American studies identified a higher risk of hospitalization in black-skinned individuals (RR 1.9; 95%CI 1.5–2.6) and among Hispanics or Latinos (RR 1.3; 95%CI 1.0–1.8).^2,16,20,23^ In Brazil, the highest risk of death was observed in indigenous peoples (RR 3.4; 95%CI 2.2–5.2), black-skinned individuals (RR 1.3; 95%CI 1.1–1.5), and inhabitants of the Northeast (RR 2.1; 95%CI 1.7–2.5) and North (RR 1.5; 95%CI 1.2–2.0) regions.^25,27^ The Table 4 presents the main risk factors for hospitalization, disease severity or death from COVID-19 in adolescents.

**Table 4:** Main risk factors for hospitalization, disease severity, or death from COVID-19 in adolescents.

– Dyspnea, fever, and cough are associated with severe cases and hospitalization.
– Severe cases are associated with the risk of death.
– Preexisting comorbidity increases the risk of hospitalization and death.
– Patients with chronic respiratory, neurological, cardiac, metabolic, or immunosuppressed diseases are at greater risk of hospitalization and death.
– The risk of hospitalization and death is greater with each additional comorbidity, dose-response relationship.
– Blacks, mixed race, indigenous peoples, and residents of poorer regions are risk groups.

As for disease diagnosis, the studies described clinical-epidemiological and laboratory methods. The most frequent test was the reverse transcription-polymerase chain reaction (RT-PCR) virus detection.^15,16,17–27^ Rapid tests for viral antigens and tests for immune response to viruses, such as antibody tests (serological tests), were also mentioned.^15,19,22^

## DISCUSSION

COVID-19 has a variable clinical presentation.^28,29^ In adolescents, there are reports of asymptomatic cases, cases of severe respiratory failure, and deaths.^15,22^ In this study, we discuss the clinical profile and the main associated comorbidities and outcomes in the age group from 10 to 19 years.

In adolescents, the prevalence of the disease varies between 20 and 30%; however, it may be even higher in poorer countries with low human development indexes, unknown until now, in several national and international regions.^4,6,17,22,30^ Compared to other age groups, teenagers are often less infected than the adult population. In contrast, the COVID-19 prevalence in adolescents appears to be higher than that observed in children. It is hypothesized that the transmission of the disease to adolescents occurs mainly through infected adults and household contacts, since adults are more exposed to the virus in the workplace, in transport, and on the streets and are, therefore, more frequently infected.^17,22,30^ On the American continent, 13% of all cases are in adolescents.^16,26^ Nearly 80% of those infected are asymptomatic or mild cases, and 20% are severe.^30^ Pinto Júnior et al. verified that asymptomatic cases account for 76% of those infected.^22^ However, the study by Afonso et al. observed that 45% of the adolescents are asymptomatic and that, among the symptomatic, most are cases of flu syndrome.^17^ In this review, the proportion of symptomatic patients varies from 40 to 70%.^17,19,22,23^ However, there is still no consensus in the literature on the prevalence of symptomatic and asymptomatic cases, considering that global testing in adolescents is not usual.^22,23,26^

The most common clinical manifestations in mild cases are headache (42%), cough (41%), fever (35%), and myalgia (30%), according to Fiocruz.^30^ Maciel et al. showed that cough (40%) and fever (26%) are prevalent symptoms and data from the United States Centers for Disease Control and Prevention reinforce what was found, mentioning fever in 56% and cough in 54% of cases.^31^ Likewise, Xia et al. cited the occurrence of cough and fever in 65% and 60%, respectively, of the infected adolescents.^32^ In the current study, fever are predominant in severe and critical cases, in 80%. However, at the beginning of the infection, fever may be low or absent, manifesting itself only days after contamination.^15,22,24,30^ Cough may appear in mild cases, from 10 to 70%, but it is higher in severe cases, with approximately 70%.^20,27^ In non-hospitalized adolescents, anosmia, nasal congestion, and ageusia are also recurrent, and nausea, vomiting, diarrhea, abdominal pain, myalgia, arthralgia, and fatigue have variable frequencies.^17,20,22,30^ In severe cases, adolescents may present SARS; this condition increases the probability of ICU admission, MV use, and death.^21,23,27,30^ There are also reports of cases that develop into pediatric multisystemic inflammatory syndrome (P-MIS).^7^ In the analysis of the articles, it is noticed that the symptoms associated with the need for hospitalization are fever, cough, dyspnea, and peripheral oxygen saturation (SpO_2_) <95%;^21,27^ of which dyspnea, cough, and fever increased the risk of ICU admission, use of MV, and death.^21,23,27^ Although children, teenagers, and adults have similar symptoms of COVID-19, children and teenagers often have a less severe infection than adults.^17,22^ While signs and symptoms of upper and lower respiratory system involvement are frequent in adolescents and adults, gastrointestinal symptoms are frequently present in children.^15,23,24^ Studies available to date show that lower airway involvement in COVID-19 infection appears uncommon in children; on the contrary, skin rashes and difficulty eating or inappetence are more prevalent. However, even if they are more asymptomatic, children and adolescents can develop P-MIS and SARS associated with COVID-19, manifesting a severe form of the disease, implying the need for hospital care, local availability of medical materials and equipment, and trained teams in disease management.^15,19,21,23,27^

Similar to adults and children, adolescents with comorbidities are more vulnerable to severe disease.^30^ Thus, strategies to combat the pandemic must target this most vulnerable population, in all age groups.^20–23^ However, age is already a significant predictive factor for a higher occurrence of severity and mortality from COVID-19, with the elderly with chronic disease being more susceptible to severe form; consequently, high mortality rates are observed in this age group.^20,27,30^ A meta-analysis found severe COVID-19 in 5.1% of adolescents with comorbidities and in 0.2% of those without comorbidities (RR 1.8; 95%CI 1.3–2.5).^30^ The presence of comorbidities also increases death risk and length of stay, regardless of age.^19,20,23,27^ According to data from the Brazilian Ministry of Health, 6% of adolescents who died from COVID-19 and 63% of those who needed invasive ventilatory support in 2021 had some comorbidity.^6^ Patients with chronic pulmonary, metabolic, neurological, cardiac, and immunosuppressive diseases are more likely to be hospitalized and experience complications from COVID-19.^23,26,27,31^ In many of these diseases, chronic inflammation, poor immune response, and underlying cardiorespiratory pathologies contribute to the need for hospitalization and the worsening of cases.^16,32,33^

According to some surveys, contamination by COVID-19 is prevalent among black, mixed-race, and indigenous peoples, as well as among inhabitants of the poorest regions worldwide.^17,22,25,26,27^ The accentuated inequalities and socioeconomic vulnerabilities in many countries may have impacted the access to information, the lack of protective equipment, and the difficulty in accessing health services.^26,27,34^ Likewise, the need for income led many adults and adolescents to the front lines during the pandemic, as many of them worked in essential services (supermarkets, bakeries, delivery services), exposing themselves more to contamination. It is evident that the living conditions affected them in a way that made them more exposed to illness and death.^30,35^ The scenario of social inequality is also repeated in other age groups since the mortality of children by COVID-19 is more common in poor countries; about 92% of global deaths from COVID-19 among children and adolescents occurred in low- and middle-income countries.^36,37^

Scientific evidence shows that the effective way to prevent severe COVID-19 is vaccination.^30^ It is imperative that adolescents receive the complete immunization schedule, as vaccine is 94% (95%CI 90–96) effective in preventing hospitalization and 98% (95%CI 93–99) effective in avoiding ICU admissions and need for life support.^34,36^ There is also the possibility that the vaccine reduces the risk of sequelae, P-MIS, and Long COVID.^30,36^ Vaccination also reduces the number of severe cases and deaths in adults and children, while minimizing disease transmission. Thus, vaccinating children and adults interferes with the indirect protection of the adolescent population, as it will increase vaccination coverage and decrease the circulation of the virus and its variants, decreasing secondary cases or possible new cases.^36,37^

It is noticeable that the number of new cases and deaths due to COVID-19 are decreasing globally, although the pandemic is not over. A new variant, more virulent and transmissible, can appear at any time; hence the importance of preventive measures.^30,34,36^ Therefore, in high-incidence regions, adolescents should be advised to avoid crowds and use masks, especially those with preexisting diseases.^34^ Besides, due to the flow of various types of respiratory viruses, associated with the low sensitivity of self-tests, and the high cost and scarcity of laboratory exams, frequent testing of symptomatic adolescents is unfeasible in some countries.^30^ Thus, any symptomatic case identified should be isolated and monitored. Also, the management of adolescent health care in the context of the COVID-19 pandemic should be performed based on the training of primary care teams since most positive cases are mild or moderate, enabling these teams to address them. In addition, it is necessary to develop a set of effective interventions for the adolescent community, expanding health education and encouraging vaccination.^30,33,36^

The current systematic review was carried out based on observational studies, which can be considered a research limitation. However, the evaluation of the methodological quality of the articles was conducted according to the recommended methods for this type of study. Hence, the research contributed to a better understanding of the profile of adolescents affected by COVID-19, fostering other studies on the topic.

The results show a possible clinical profile of COVID-19, associated comorbidities, and outcomes in adolescents. Fever, cough, headache, anosmia, nasal congestion, and ageusia were prevalent in mild cases. In hospitalized patients, fever and cough were more frequent, as well as dyspnea and SpO_2_ <95%. There was an association between previous comorbidities and disease severity, including a dose-response relationship, increasing hospitalization risk, need for intensive care, use of MV, and death. Contamination is prevalent in black, mixed-race, and indigenous people, as well as in inhabitants of poorer regions. The study permitted a better understanding of the disease profile in adolescents. Thus, it can contribute to the elaboration of public health policies and interventions, and to current literature in the field of adolescent health.
