# ERRATUM

**DOI:** 10.1590/1984-0462/2024/42/2022241erratum

**Published:** 2024-02-02

In the manuscript “Characteristics and clinical outcomes of adolescents infected by SARS-CoV-2: a systematic review”, DOI: 10.1590/1984-0462/2024/42/2022241, published in the Rev Paul Pediatr. 2024;42:e2022241, on page 3:

**Where it reads:**

**Table 1.** Articles published between January 2020 and April 2022.

Reference	Country	Study design	Description of the sample Number of participants (n)/age group (years old)	Methodological quality^14^ and level of evidence^12^
Oliveira et al.^27^	Brazil	Cohort	n=6.725/12-19	17 points/level 3
Clemente et al.^19^	Spain	Cohort	n=77/less than 18	16 points/level 3
Drouin et al.^20^	Canada	Cohort	n=36/13-17	18 points/level 3
Gomes et al.^21^	Brazil	Cohort	n=1320/12-18	16 points/level 3
Pinto Júnior et al.^22^	Brazil	Cross-sectional	n=854/10-19	17 points/level 4
Graff et al.^23^	USA	Cohort	n=220/11-19	17 points/level 3
Macias-Parra et al.^24^	Mexico	Cross-sectional	n=34/12-18	16 points/level 4
Oliveira et al.^25^	Brazil	Cohort	n=3589/12-19	21 points/level 3
Parcha et al.^26^	USA	Cohort	n=5573/12-17	18 points/level 3
Prata-Barbosa et al.^15^	Brazil	Cohort	n=14/12-17	16 points/level 3
DeBiasi et al.^16^	USA	Cohort	n=73/10-19	16 points/level 3

**It should read:**

**Table 1.** Articles published between January 2020 and April 2022.

Reference	Country	Study design	Description of the sample Number of participants (n)/age group (years old)	Methodological quality^14^ and level of evidence^12^
Oliveira et al.^27^	Brazil	Cohort	n=6.725/12-19	17 points/level 3
^Afonso et al.17^	Brazil	Cross-sectional	n=49/10-19	17 points/level 4
^Alharbi et al.18^	Saudi Arabia	Cohort	n=9/11-14	16 points/level 3
Clemente et al.^19^	Spain	Cohort	n=77/less than 18	16 points/level 3
Drouin et al.^20^	Canada	Cohort	n=36/13-17	18 points/level 3
Gomes et al.^21^	Brazil	Cohort	n=1320/12-18	16 points/level 3
Pinto Júnior et al.^22^	Brazil	Cross-sectional	n=854/10-19	17 points/level 4
Graff et al.^23^	USA	Cohort	n=220/11-19	17 points/level 3
Macias-Parra et al.^24^	Mexico	Cross-sectional	n=34/12-18	16 points/level 4
Oliveira et al.^25^	Brazil	Cohort	n=3589/12-19	21 points/level 3
Parcha et al.^26^	USA	Cohort	n=5573/12-17	18 points/level 3
Prata-Barbosa et al.^15^	Brazil	Cohort	n=14/12-17	16 points/level 3
DeBiasi et al.^16^	USA	Cohort	n=73/10-19	16 points/level 3

